# Routine blood tests are associated with short term mortality and can improve emergency department triage: a cohort study of >12,000 patients

**DOI:** 10.1186/s13049-017-0458-x

**Published:** 2017-11-28

**Authors:** Michael Kristensen, Anne Kristine Servais Iversen, Thomas Alexander Gerds, Rebecca Østervig, Jakob Danker Linnet, Charlotte Barfod, Kai Henrik Wiborg Lange, György Sölétormos, Jakob Lundager Forberg, Jesper Eugen-Olsen, Lars Simon Rasmussen, Morten Schou, Lars Køber, Kasper Iversen

**Affiliations:** 1 Department of Emergency Medicine, Sjællands Universitetshospital Køge, Køge, Denmark; 2 Department of Cardiology, Endocrinology and Nephrology, Nordsjællands Hospital, Copenhagen University Hospital, Copenhagen, Denmark; 3 Department of Cardiology, Herlev Hospital, Copenhagen University Hospital, Copenhagen, Denmark; 4 Department of Biostatistics, University of Copenhagen, Copenhagen, Denmark; 5 Department of Anaesthesia, Sjællands Universitetshospital Køge, Køge, Denmark; 6 Department of Anaesthesia, Centre of Head and Orthopaedics Surgery, Rigshospitalet, University of Copenhagen, Copenhagen, Denmark; 7 Department of Anaesthesia, Nordsjællands Hospital, Hillerød, Denmark; 8 Department of Research, Nordsjælland Hospital, Hillerød, Denmark; 9 Department of Prehospital and In-Hospital Emergency Medicine – Helsingborg Hospital, Helsingborg, Sweden; 10 Clinical Research Centre, Copenhagen University Hospital, Hvidovre, Denmark; 11 Department of Cardiology, Rigshospitalet, University of Copenhagen, Copenhagen, Denmark

**Keywords:** Triage, Biomarkers, Risk stratification, Emergency medicine, Acute patients

## Abstract

**Background:**

Prioritization of acutely ill patients in the Emergency Department remains a challenge. We aimed to evaluate whether routine blood tests can predict mortality in unselected patients in an emergency department and to compare risk prediction with a formalized triage algorithm.

**Methods:**

A prospective observational cohort study of 12,661 consecutive admissions to the Emergency Department of Nordsjælland University Hospital during two separate periods in 2010 (primary cohort, *n* = 6279) and 2013 (validation cohort, *n* = 6383). Patients were triaged in five categories by a formalized triage algorithm. All patients with a full routine biochemical screening (albumin, creatinine, c-reactive protein, haemoglobin, lactate dehydrogenase, leukocyte count, potassium, and sodium) taken at triage were included. Information about vital status was collected from the Danish Central Office of Civil registration.

Multiple logistic regressions were used to predict 30-day mortality. Validation was performed by applying the regression models on the 2013 validation cohort.

**Results:**

Thirty-day mortality was 5.3%. The routine blood tests had a significantly stronger discriminative value on 30-day mortality compared to the formalized triage (AUC 88.1 [85.7;90.5] vs. 63.4 [59.1;67.5], *p* < 0.01). Risk stratification by routine blood tests was able to identify a larger number of low risk patients (*n* = 2100, 30-day mortality 0.1% [95% CI 0.0;0.3%]) compared to formalized triage (*n* = 1591, 2.8% [95% CI 2.0;3.6%]), *p* < 0.01.

**Conclusions:**

Routine blood tests were strongly associated with 30-day mortality in acutely ill patients and discriminatory ability was significantly higher than with a formalized triage algorithm. Thus routine blood tests allowed an improved risk stratification of patients presenting in an emergency department.

**Electronic supplementary material:**

The online version of this article (10.1186/s13049-017-0458-x) contains supplementary material, which is available to authorized users.

## Background

Prioritization of acutely ill patients in the Emergency Departments (ED) remains a challenge. Waiting time to treatment is not only the most important predictor of patient satisfaction [1], it can also lead to poor outcome [2, 3].

Risk stratification of patients in the Emergency Department using triage systems has been proposed to meet the above mentioned challenges [1]. Typically, these triage systems classify patients into five categories based on vital signs (level of consciousness, arterial blood pressure, heart rate, arterial oxygen saturation, respiratory rate and temperature) [4, 5]. In recognition that the severity of acute illness cannot be recognized solely on the vital signs most triage models upgrade patients in certain high risk situations to higher levels of urgency based on presenting complaint. [1] This leads to lower specificity and potentially an inappropriate allocation of resources adversely affecting other patients in the ED [6–9].

A routine biochemical screening is performed on nearly all patients upon presentation to the modern ED except for those with obvious minor complaints. The results of these blood tests are, however, rarely used in the initial risk assessment even though modern blood analysis techniques allow for the results to be available within 15 min. Many routine tests have a well-known prognostic value in selected patient groups, but their value for initial risk assessment of ED patients have to our knowledge not been investigated in larger groups [10–14].

The aim of this study was to investigate, if routine blood tests could improve the prediction of outcome in unselected patients in the emergency department as compared with a formalized triage algorithm.

## Methods

The study was a prospective observational cohort study of 12,661 consecutive admissions at the Emergency Department of Nordsjælland University Hospital over two periods in 2010 (primary cohort, *n* = 6249) and 2013 (validation cohort, *n* = 6383). Initial data analyses were performed on data from the primary cohort. Data on demographics and routine blood test results were analysed with logistic regression statistics to create a model for predicting mortality. As low and high values of any blood test may increase risk of mortality equally (e.g. hypokalaemia and hyperkalaemia) relevant variables were modelled by splines rather than linearly. The prediction model was ultimately validated on the 2013 cohort and predictive ability was compared to the original triage.

### Study data

The ED of Nordsjælland University Hospital is one of the largest regional hospitals in the Capital Region of Denmark. The hospital has a 24-h acute care facility offering emergency, level-2 trauma, medical, surgical and intensive care services for 310,000 citizens. The ED has approximately 68,000 patient contacts annually and complete regional uptake, eliminating selection due to hospital preferences by the patients [15].

The primary cohort was based on the ‘Acute Admission Database’ comprising 6279 consecutive patients referred or presented to the ED from September 22, 2009 to February 28, 2010. Inclusion criteria were age > 16 years and presentation to the ED. The validation cohort was based on the ‘Triage Database’ comprising 6383 consecutive patients presenting to the ED from September 4 to December 13, 2013. Patients <17 years and obstetrics patients were not included. Patients detected in the field with major trauma, ST-elevation acute myocardial infarction or stroke within 2–3 h were admitted to the tertiary centre in the region. Patients with minor complaints and injuries planned for ambulant or fast track treatment were excluded (i.e. no patients categorized at the lowest level of urgency were included). Both studies have previously been described in details [5, 16].

For this study, we included patients with a full routine biochemical screening (albumin, creatinine, c-reactive protein, haemoglobin, lactate dehydrogenase, leukocyte count, potassium, and sodium) and vital status.

All patients in the two cohorts were identified by the Central Personal Registry (CPR) number that is assigned to all Danish citizens at birth or immigration and this uniquely identifies gender and date of birth. Information about vital status was obtained from The Danish Central Office of Civil Registration that records the vital status of all Danish residents.

### Triage

Nordsjælland University Hospital started using the Hillerød Adaptive Process Triage (HAPT) in 2009 inspired by the Swedish Adaptive Process Triage model (ADAPT) [5]. ADAPT is a five-level triage system, ranking the patients based on both vital signs and presenting complaint. Patients are triaged as red (life-threatening), orange (seriously ill), yellow (ill), green (need of assessment) or blue (minor complaints). The most urgent of vital signs or presenting complaint determines the final triage category [5, 17]. In 2011 HAPT was customized for local conditions and named Danish Emergency Process Triage (DEPT) [18]. This system is the most widely used triage system in Denmark [19, 20]. For details on the DEPT triage system see Additional file 1.

### Statistics

Multiple logistic regression was used to predict the primary endpoint, 30-day mortality. Four models were compared to triage: One including the eight routine blood tests, one with demographics (age and sex), one with blood tests and demographics, and one including the routine blood tests, demographics and formalized triage.

The effect of the eight blood tests on the log odds of 30-day mortality was assumed to be linear or modelled by a linear spline for which change points were chosen according to recommended age-sex specific normal reference values (see Additional file 2 for details) [21]. We also developed a univariate logistic regression model based on the four triage levels.

From the multiple logistic regression models, we calculated the predicted risks of 30-day mortality on the probability scale. Based on the predicted risks of 30-day mortality patients were reclassified into four groups (green <1%, yellow 1–10%, orange >10–25%, red >25%) in order to match the four triage levels. Survival probabilities within the first 30 days were calculated in risk strata by Kaplan-Meier method.

The diagnostic accuracy of the logistic regression models was evaluated on discriminative value and calibration. The discriminative value is the ability of a model to differentiate between two conditions, e.g. low risk or high risk. Calibration can be defined as the accuracy of the prediction. E.g. if a model predicts a risk of x/100 we can expect x/100 patients to have the event [22].

The discriminative value of the logistic regression models was evaluated using receiver operation characteristics curves (ROC) and the area under the curve (AUC). AUC can be interpreted as the probability that a logistic regression model will predict a randomly chosen patient who died within 30 days a higher risk than a randomly chosen patient who survived 30 days. A high AUC indicates good discriminative value [23]. Being a rank statistic AUC is invariant to monotone transformation of the predicted risks and hence cannot indicate failure to model calibration. To asses model calibration Brier scores were obtained and calibration plots created. The Brier score is the mean squared difference between the predicted probability of 30-day mortality and the actual outcome for this patient. The lower the Brier score, the better the prediction accuracy [24, 25].

The results were internally validated by a cross-validation approach using 1000 splits of the data into training and validation set [26]. We report the mean AUC and the 2.5% and the 97.5% quantiles of 1000 cross-validation results.

External validation was performed by applying the regression models built on the primary cohort to the 2013 cohort and calculating AUC and Brier score. The 30-day mortality risk predictions based on the blood test prediction model were re-calibrated for the validation cohort using logistic calibration described by Janssen et al. 2008 [27].

All data management and statistical computation was performed using the R software version 3.1.0 [28–30].

## Results

### Primary cohort

A full biochemical screening, triage category, and vital status were available for 85.5% of the patients in the primary 2010 cohort. Characteristics of the included and excluded patients are presented in Additional file 3 along with vital signs and blood test results. The excluded group differed significantly from the included concerning the following variables: They were younger, had a higher rate of admission to ICU, they had shorter length of stay, they more frequently presented during weekends and during the night, and finally, they had a higher arterial oxygen saturation and a lower respiratory rate.

### Validation cohort

Triage category, vital status and a full biochemical screening were available for 5738 of the 6383 (89.9%) patients in the 2013 validation cohort (Table 1). The validation cohort was collected in the same ED as the primary cohort however three years later the hospital managed a larger number of annual contacts due to the closing of a smaller local hospital. 30-day mortality was significantly lower than in the primary cohort (4.1% vs. 5.3%, *p* < 0.01). They had a lower respiratory rate, lower heart rate, and lower systolic blood pressure. The levels of albumin, c-reactive protein, potassium and leukocyte count were lower. Sodium level was higher. Se cohorts compared in Additional file 4.

**Table 1:** Characteristics of included patients admitted to the emergency department in primary cohort (2010) and validation cohort (2013)

	Primary cohort (*n* = 5371)	Validation cohort (*n* = 5738)	*p*-value
Age (median, IQR)	63.8 [46.92–76.52]	63.0 [46.0–76.0]	0.21
Male gender (n, %)	2578 (48.0)	2835 (49.4)	0.14
30-day mortality (n, %)	284 (5.3)	234 (4.1)	<0.01
48-h mortality (n, %)	61 (1.1)	50 (0.9)	0.19
C-reactive protein, nmol/L (median, IQR)	5.8 [1.7–29.17]	4.9 [2.9–22.2]	<0.001
Potassium, mmol/L (median, IQR)	4.1 [3.8–4.4]	4.0 [3.9–4.3]	<0.001
Sodium, mmol/L (median, IQR)	137.5 [135.0–139.4]	139 [136.9–140.7]	<0.001
Haemoglobin, mmol/L (median, IQR)	8.4 [7.6–9.1]	8.4 [7.6–9.1]	0.52
Creatinine, μmol/L (median, IQR)	71.2 [59.0–87.0]	71.0 [60.0–87.0]	0.77
Leukocyte count, 109/L (median, IQR)	8.7 [6.8–11.5]	8.2 [6.5–10.6]	<0.001
Albumin, g/L (median, IQR)	42.0 [38.6–44.7]	37.2 [33.6–39.8]	<0.001
Lactate dehydrogenase, U/L (median, IQR)	178.1 [153.3–213.6]	182.2 [157.0–216.9]	0.50
Arterial oxygen saturation, % (median, IQR)	98 [96–99]	98 [96–99]	0.31
Respiratory rate, min^−1^ (median, IQR)	16 [16–20]	16 [15–19]	<0.001
Heart rate, min^−1^ (median, IQR)	82 [71–95]	80 [70–92]	<0.001
Systolic blood pressure, mmHg (median, IQR)	140 [125–157]	134 [119–150]	<0.001

### Main results

There was a significant association between 30-day mortality and all eight blood tests as well as age in the individual linear models. However in the final model - including all eight blood tests, age and sex - a significant association with mortality was found only for albumin, creatinine, c-reactive protein, potassium, lactate dehydrogenase, age, and sex.

#### Discriminative ability

**Fig. 1:**
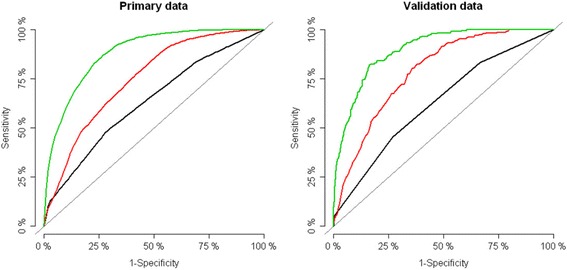
Discriminative abilities in relation to 30 day-mortality. Black, primary data: HAPT triage, AUC = 63.4%. Red, primary data: Demographics (age + sex), AUC = 74.9%. Green, primary data: Routine blood tests, AUC = 86.2%. Black, validation data: DEPT triage, AUC = 62.8%. Red, validation data: Demographics (age + sex), AUC = 78.5%. Green, validation data: Routine blood tests, AUC = 86.7%. Receiver operation characteristics showing discriminative value of triage, demographics and routine blood tests^a^ for prediction of short term mortality. Primary cohort (left) and validation cohort (right). a: Albumin, creatinine, c-reactive protein, haemoglobin, lactate dehydrogenase, leukocyte count, potassium, sodium

Discriminative abilities in relation to 30 day-mortality are visualised with ROC-curves (Fig. 1, panel A: Primary cohort, B: Validation cohort). AUC-values and Brier scores with 95% confidence intervals are presented in Table 2. Demographics alone (age and sex) was significantly stronger than the original triage (panel A, red vs. black line) AUC 74.9% [95% CI = 71.4;78.2] vs. 63.4% [95% CI = 59.1;67.5], *p* < 0.01. The routine blood tests had a significantly stronger discriminative value than triage and demographics (panel A, green line) AUC 86.2% [95% CI = 83.1;89.3], *p* < 0.01. Adding demographics to the blood test prediction model had no significant effect (AUC 88.1 [95% CI = 85.7;90.5]). Applying the prediction model on the validation cohort yielded similar results. The blood test prediction model was significantly stronger than the formalized triage (DEPT) (AUC = 87.7 [95CI = 84.3;88.5] vs. AUC = 62.8% [95CI = 59.1;65.7], *p* < 0.01).

**Table 2:** Area under the receiver operation characteristics curve and Brier score for prediction of 30-day mortality in patients presenting in the emergency department

	Primary cohort (*n* = 5371)^a^		Validation (*n* = 5738)^b^	
	AUC, % [95% CI]	Brier score, % [95% CI]	AUC, % [95% CI]	Brier score, % [95% CI]
Triage	63.4 [59.1;67.5]	4.90 [4.24;5.62]	62.8 [59.4;66.2]	3.85 [3.40;4.30]
Demographics^c^	74.9 [71.4;78.2]	4.79 [4.17;5.49]	78.5 [76.1;81.0]	3.75 [3.34;4.17]
Blood tests^d^	86.2 [83.1;89.3]	4.22 [3.62;4.82]	86.7 [84.4;88.9]	3.77 [3.44;4.11]
Blood tests^d^ + demographics^c^	88.1 [85.7;90.5]	4.18 [3.60;4.79]	89.7 [88.1;91.4]	3.56 [3.24;3.88]
Blood tests^d^ + demographics^c^ + triage	88.6 [86.1;91.1]	4.11 [3.54;4.70]	90.8 [89.2;92.3]	3.40 [3.08;3.72]

^a^HAPT-triage. ^b^DEPT-triage. ^c^Age and sex. ^d^Albumin, creatinine, c-reactive protein, haemoglobin, lactate dehydrogenase, leukocytes, potassium, sodium

#### Calibration

Though discriminatory value was good, the blood test prediction model systematically overestimated the risk of 30-day mortality when applied to the validation cohort. Hence poor calibration. After recalibration of the model, we saw a predictive accuracy as in the primary cohort. This is illustrated in Table 3 showing distribution of patients listed according to: 1) Formalized triage, 2) Blood test prediction model before recalibration and 3) Blood test prediction model after recalibration. Further notes and details on recalibration in Additional file 5.

**Table 3:** Distribution of patients by formalized triage and blood test prediction model

	Validation cohort (*n* = 5738)		
	DEPT	Blood test prediction^a^ No calibration	Blood test prediction^a^ Recalibrated
Green	1876 (2.1% [1.4;2.7])	235 (0% [0;0])	2030 (0.4% [0.1;0.7])
Yellow	2272 (3.9% [3.1;4.7])	4205 (1.3% [0.9;1.6])	3174 (3.3% [2.7;3.9])
Orange	1557 (6.0% [4.9;7.2])	821 (8.0% [6.2;9.9])	365 (15.1% [11.4;18.7%])
Red	33 (36.4%[20.0;52.8%])	477 (23.9% [20.1;27.7])	169 (39.6% [32.3;47.0])

Distribution of patients, risk stratified by formalized triage algorithm (DEPT) or the blood test prediction model. 30-day mortality with 95% confidence intervals for individual strata in parenthesis. Initially blood test prediction model overestimates risk of mortality, seen by large number of high risk patients. After recalibration. ^a^Predicted risk of 30-day mortality: green <1%, yellow 1–10%, orange 10–25%, red >25%

**Fig. 2:**
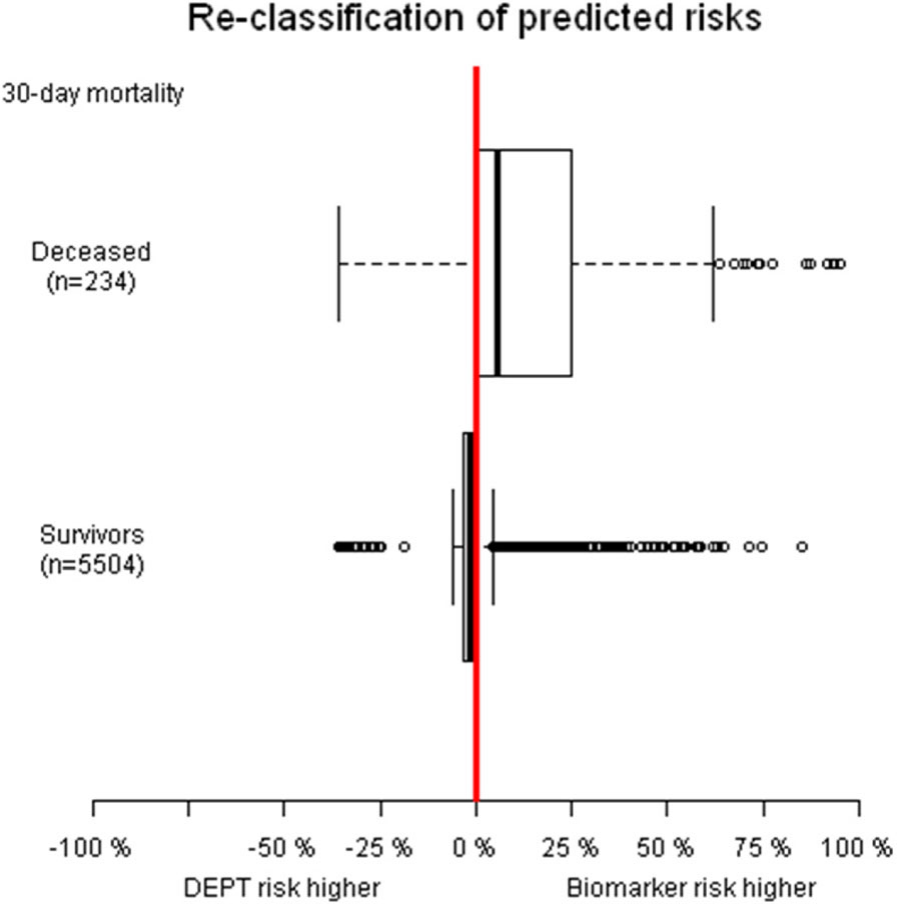
Boxplots of differences in predictive risks conditional on 30-day vital status. DEPT triage of patients presenting in the emergency department compared to the blood test prediction model applied on the validation cohort

**Fig. 3:**
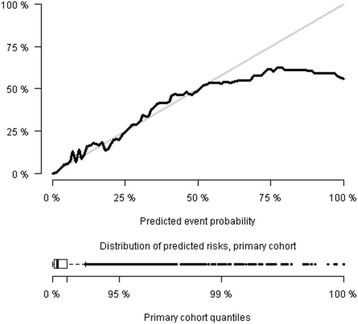
Calibration plot of the blood test prediction model applied on the primary cohort. The prediction model includes the 8 blood tests and demographics (age and sex). Distribution of predicted risks are illustrated with boxplot. Black: Predicted risks

**Fig. 4:**
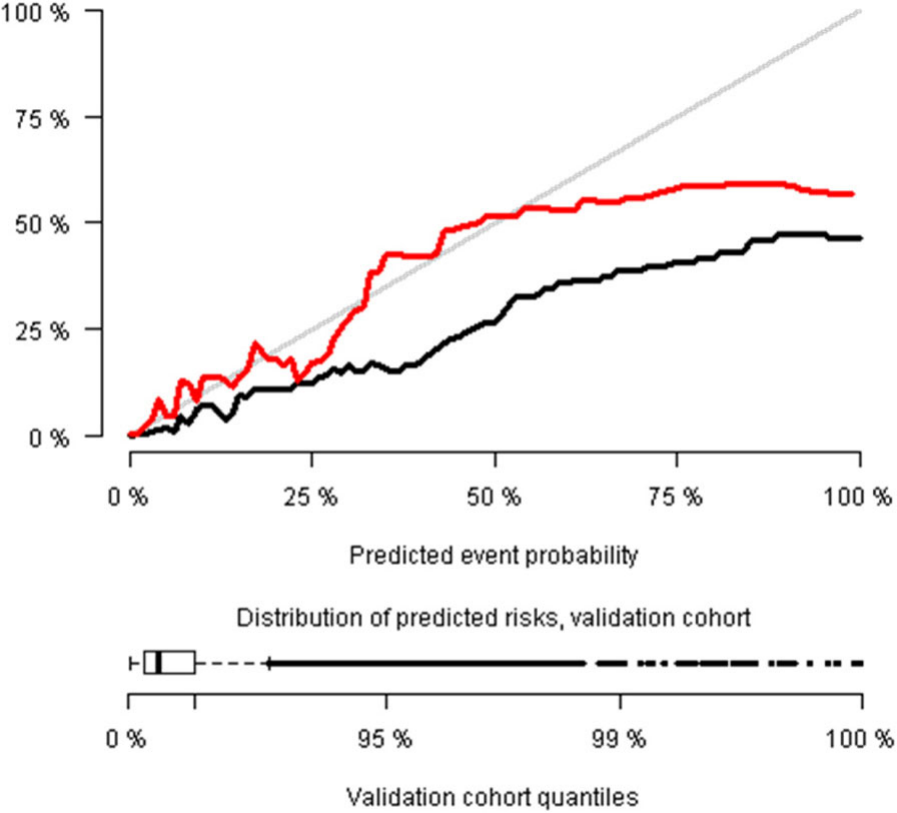
Calibration plot of the blood test prediction model applied on the validation cohort. Black line is before recalibration. Red line is after recalibration. The prediction model includes the 8 blood tests and demographics (age and sex). Distribution of predicted risks are illustrated with boxplot in the bottom. Black: Predicted risks, no recalibration. Red: Predicted risks, after recalibration

Figure 2 illustrates the stronger predictive accuracy of the blood test prediction model compared to formalized triage (DEPT). Calibration plots of the blood test prediction model on primary and validation cohort (Figs. 3 and 4) illustrates equivalent calibration (i.e. model fit) on both cohorts.

## Discussion

We found that routine blood tests can accurately predict 30-day mortality and improve risk stratification of patients admitted to the Emergency Department. The eight routine biochemical measurements provided a significantly stronger discriminative ability with respect to 30-day mortality compared to the formalized triage algorithm and we were able to validate the findings on a secondary cohort. In particular, the blood test prediction model seems to be valuable with respect to identifying low risk patients, which is interesting as triage model have been assessed by focusing on mortality among patients graded as low risk [31].

Currently used formalized triage have been validated to a limited extent [19, 31]. Level of consciousness, arterial oxygen saturation, and respiratory rate - variables included in many modern triage systems [7, 31–33] - are significant individual predictors of mortality during hospitalization [31, 34] but no studies have reported valid scientific evidence for the triage scales to predict mortality when adjusted for age [35]. In this study the eight routine blood tests were found to be strongly associated with 30-day mortality independent of age and sex.

The idea of using blood tests in the clinical setting for risk stratifying or as an aid in decision-making is not new. Biochemical analyses are routinely used in various ICU scoring systems, e.g. the APACHE II system that is validated for use in surgical as well as general ICU patients. The APACHE II system uses haematocrit, potassium, leukocyte count, sodium, and arterial pH along with level of consciousness and clinical values [9]. APACHE II also includes age as this is a well-documented risk factor for death from acute illness independent of the severity of disease [34]. Though previous studies have proposed, that including blood tests into the ED risk stratification models may add significant predictive value [9, 10], such an ED scoring system is to our knowledge still missing.

Some limitations of using biochemical measurements in ED triage must be pointed out. First of all, we needed to recalibrate the model to get good calibration when applying it on the validation cohort (Figs. 3 and 4). This may be explained by differences in patient characteristics between the two cohorts. Just as formalized triage algorithms are adjusted for local conditions, a biochemical prediction model may need calibration due to local differences, such as in demographics, biochemical assays used or maybe even seasonal changes. However we have shown, that the model can easily be recalibrated to local conditions if needed.

Another limitation of a triage system using blood tests is the delay associated with the analysis of blood samples. However with a modern automated laboratory a full package of routine blood sample results can be ready within 15 min and even sooner with point-of-care methods. Finally, one must consider that some acute conditions may not be detectable at admission by blood sample analysis, for instance myocardial infarction or stroke. In addition, some biochemical variables, e.g. albumin and creatinine, may be related to a chronical disease rather than an acute condition. Hence, the blood tests should not stand alone. Rather they may be useful in combination with vital signs on admission as well as with age and sex.

An easy way to apply the results from the routine blood tests in a clinical setting would be through a simple computer algorithm. A predicted risk of short term mortality for each patient could be delivered automatically with the blood test results or computed afterwards in a software for computer or mobile phone. This could be an aid for the physician when risk stratifying and prioritizing in the ED.

A strength of the present study is the large sample size and a relatively large number of events (approximately 5% mortality within 30 days). Furthermore, the data comprise a combination of surgical as well as non-surgical patients reflecting the real world ED. In this study we have chosen to compute a predicted risk of mortality based on the blood tests with a logistic regression model with splines. A simpler approach would be to count the number of blood tests out of the reference area as done in other scoring systems. However we decided to use a more complex method to maximise the predictive potential of the routine blood tests. The regression splines also add flexibility to the model and allow for low and high values of a given blood test to have an impact on the predicted risk. Demographics (age and sex) can easily be included in the model and thereby improve accuracy. Finally as no backwards elimination was done, i.e. all eight blood tests were included, we have challenged our model maximally. The discriminatory abilities were assessed with ROC curves and AUC after an internal cross validation with 1.000 bootstrap samples. Furthermore external validation of the model using a new large cohort of patients confirmed the strength of the model.

A weakness of this study is that 30-day mortality may not always reflect the severity of the patient’s condition at admission. When risk stratifying for the purpose of immediate treatment, one could argue that a more short-term endpoint could be used, e.g. 48 h mortality. However many acute conditions can have an effect on mortality in a period beyond 48 h. Events such as ICU admission, urgent surgery and thrombolysis definitely identify high-risk patients but they are relatively rare and can never stand alone as endpoint in a population as diverse as in the ED. Any consensus on a “gold standard” for ED triage endpoints remains undetermined in the literature, but from an overall perspective 30-day mortality is a strong endpoint.

It is also important to realise that adequate treatment will affect outcome and result in a lower mortality as the information was available to the clinician. The study therefore focused on those patients who died in spite of treatment.

## Conclusion

In conclusion, the investigated routine blood tests were strongly associated with 30-day mortality in patients presenting at the Emergency Department and they allowed significantly more accurate prediction than a formalized triage algorithm. Thus, incorporating these results in the early risk stratification, could be an important help for the ED physician when prioritization of patients is needed.

## Additional files

Additional file 1: DEPT Triage. Algorithm for DEPT Triage. (DOCX 18 kb)

Additional file 2: Multivariate logistic regression model. Details on the multivariate logistic regression model and linear splines. (DOCX 79 kb)

Additional file 3: Primary Cohort. Characteristics of included and excluded patients admitted to the emergency department in primary cohort (2010). (DOCX 16 kb)

Additional file 4: Cohort Characteristics. Characteristics of the primary cohort (2010) and the validation cohort (2013). (DOCX 17 kb)

Additional file 5: Recalibration. Notes on the recalibration process. Including table depicturing reclassification of patients by the blood test prediction model and Kaplan Meier survival probabilities. (DOCX 38 kb)

## Abbreviations

APACHE II: Acute Physiology and Chronic Health Evaluation. A severity of disease classification system for use in intensive care units
AUC: Area Under the Curve
DEPT: Danish emergency process triage. Triage system developed in Denmark. Based on Hillerød adaptive triage system
ED: Emergency Department
HAPT: Hillerød adaptive process triage. Triage system developed in Hillerød, Denmark. Based on the Swedish Adaptive Process Triage model (ADAPT)
ICU: Intensive Care Unit
ROC: Receiver Operation Characteristics
