# Supplementary material for: Routine blood tests are associated with short term mortality and can improve emergency department triage: a cohort study of >12,000 patients
Source: Scand J Trauma Resusc Emerg Med. 2017 Nov 28;25:115. doi: 10.1186/s13049-017-0458-x (PMC5704435; doi:10.1186/s13049-017-0458-x)
Supplement: Supplementary file 1 — DEPT Triage. Algorithm for DEPT Triage. (DOCX 18 kb) [file 13049_2017_458_MOESM1_ESM.docx]

# DEPT Triage

## Blue (Fast track)

If “Yes” to all of following questions, the patient can be triaged BLUE:

1. The patient’s general condition is unaffected (respiration, colour, level of consciousness)
2. Minor injury with reasonable relationship between cause and effect
3. Injury not related to prior illness
4. Presenting complaint listed in manual

## DEPT-triage by physiological parameters

|  | Red  Resuscitation (0 min.) | Orange  Urgent (15 min.) | Yellow  Less urgent (60 min.) | Green  Not urgent (180 min.) | Blue  Fast track (240 min.) |
| --- | --- | --- | --- | --- | --- |
| A | Obstructed airway  Stridor | Threatened airway |  |  | No vital signs |
| B | SpO_2_<80%, no O_2_  SpO_2_<90%, with O_2_  RR>35 or <5 | SpO_2_ 80 to 89%, no O_2_  SpO_2_ 90 to 94%, with O_2_  RR 31 to 35 | SpO_2_ 90 to 94%, no O_2_  RR 26 to 30 | SpO_2_ ≥95%, no O_2_  RR 8 to 25 |  |
| C | Heart rate >140 and  SBP <80 | Heart rate 121 to 140  Heart rate < 40  SBT: 80 to 89 mmHg | Heart rate: 111 to 120 Heart rate: 40 to 49 | Heart rate 50 to 110 |  |
| D | GCS ≤ 8 | GCS 9 to 13 | GCS =14 | GCS = 15 |  |
| E | Tp < 32 °C | Tp > 40 °C  Tp 32.0 to 34.0 °C | Tp 38.1 to 40.0 °C  Tp 34.1 to 35.0 °C | Tp 35.1 to 38.0 °C |  |
| For patients with COPD oxygen saturation values are decreased by 5% | | | | |  |
| B | SpO_2_<75%, no O_2_  SpO_2_<85%, with O_2_ | SpO_2_ 75 to 84%, no O_2_  SpO_2_ 85 to 89%, with O_2_ | SpO_2_ 85 to 89%, no O_2_ | SpO_2_ ≥90%, no O_2_ |  |

A = airways; B = breathing; C = circulation; D = disability; E = exposure

SBP = systolic blood pressure; GCS = Glasgow Coma Scale; RR = respiratory rate;

SpO_2_ = arterial oxygen saturation (pulse oximetry); Tp = Temperature, O_2_: Oxygen,

## Example of presenting complaint triage

DEPT triage comprises 50 different symptom triage cards. Below presenting complaint 10, chest pain:

|  | Red  Life threatening (0 min.) | Orange  Urgent (15 min.) | Yellow  Less urgent (60 min.) | Green  Not urgent (180 min.) |
| --- | --- | --- | --- | --- |
| Chest pain, tightness/pressure/ discomfort in chest |  | Yes, in last 24 hours |  |  |
| Duration |  | Minutes to hours, constant | Minutes, transient |  |
| ECG abnormities |  | New ST-T changes or broad complex arrhythmia | Narrow complex arrhythmia | None |

ECG = Electro cardiogram

## Final triage

The higher of the two triages determines the final triage level. Nurses can increase triage level. Doctors can decrease level.
