# Supplementary material for: Routine blood tests are associated with short term mortality and can improve emergency department triage: a cohort study of >12,000 patients
Source: Scand J Trauma Resusc Emerg Med. 2017 Nov 28;25:115. doi: 10.1186/s13049-017-0458-x (PMC5704435; doi:10.1186/s13049-017-0458-x)
Supplement: Supplementary file 2 — Multivariate logistic regression model. Details on the multivariate logistic regression model and linear splines. (DOCX 79 kb) [file 13049_2017_458_MOESM2_ESM.docx]

# Multivariate logistic regression model

For all eight blood samples we initially created a simple univariate regression models as well as a univariate regression model with splines. The latter in order to allow for both low and high values of a blood test to have an impact of the risk of 30-day mortality.

Potassium, leukocytes and creatinine were included in the model with linear splines. For the remaining variables the correlation was linear and splines added no extra value. Hence they were included as linear variables in the final logistic regression model.

To illustrate the effect of the splines, the univariate regression models with splines for each of the eight blood tests are depicted below. Visually it can quite easily be seen, that hypokalaemia as well as hyperkalaemia have an impact on 30-day mortality (Figure 9). In a simple univariate linear regression potassium lost a great part of its predictive value.

Figures 1-15: Univariate regression models. 30-day mortality by each of the eight routine blood tests with linear splines. Cuts are defined after age-sex specific reference intervals, and individual models are shown according to age and sex when relevant.

1: Leukocyte count

2: Sodium (linear)

3-5: Albumin (linear). Age groups <40, 40-70, >70

6: C-reactive protein (linear)

7-8: Haemoglobin (linear). Grouped by sex.

9: Potassium

10-14: Creatinine. Grouped by sex and by age groups: ≤70 and >70.

15: Lactate dehydrogenase (linear)

**Figure 1: The association between leucocyte count and 30-day mortality cumulated for both genders as linear spline. Cuts are defined after recommended reference interval (3.5 – 8.8 x 10^9^/L). Top box-plot illustrates distribution of patients. Red lines marks the reference interval.**

**
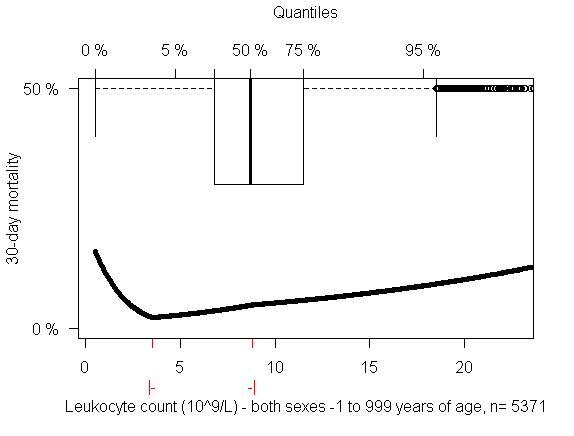
**

**Figure 2: The association between serum sodium concentration and 30-day mortality cumulated for both genders as linear spline. Cuts are defined after recommended reference interval (137-145 mmol/L). Top box-plot illustrates distribution of patients. Red lines mark the reference interval.**

**
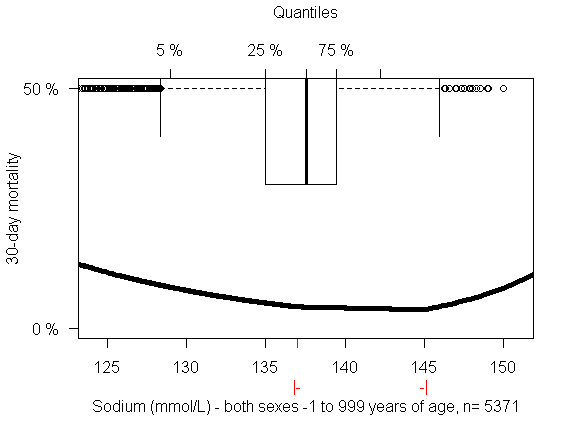
**

**Figure 3: The association between albumin concentration and 30-day mortality cumulated for both genders as linear spline for patients under 40 years of age. Cuts are defined after recommended age specific reference intervals (18-39 years: 36-48 g/L. 40-69 years: 36-45 g/L. >70 years: 34-45 g/L). Top box-plot illustrates distribution of patients. Red lines marks the reference interval.**

**
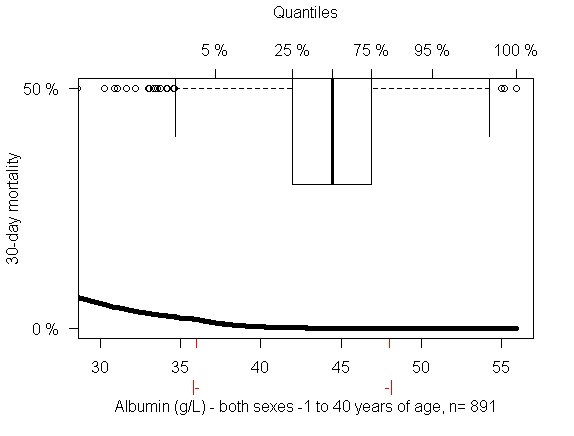
**

**Figure 4: The association between albumin concentration and 30-day mortality cumulated for both genders as linear spline for patients 40-70 years of age. Cuts are defined after recommended age specific reference intervals (18-39 years: 36-48 g/L. 40-69 years: 36-45 g/L. >70 years: 34-45 g/L). Top box-plot illustrates distribution of patients. Red lines mark the reference interval.**

**
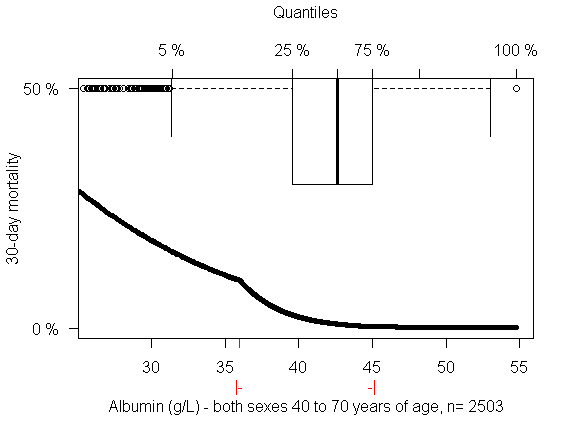
**

**Figure 5: The association between albumin concentration and 30-day mortality cumulated for both genders as linear spline for patients over 70 years of age. Cuts are defined after recommended age specific reference intervals (18-39 years: 36-48 g/L. 40-69 years: 36-45 g/L. >70 years: 34-45 g/L). Top box-plot illustrates distribution of patients. Red lines mark the reference interval.**

**
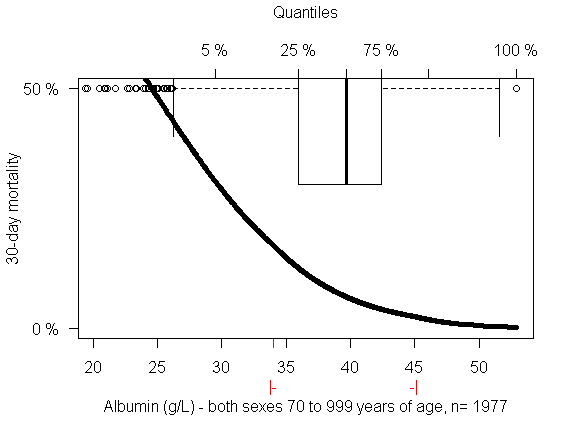
**

**Figure 6: The association between C-reactive protein concentration and 30-day mortality cumulated for both genders as linear spline. Cuts are defined after recommended reference interval (5-10 mg/L). Top box-plot illustrates distribution of patients. Red lines mark the reference interval.**

**
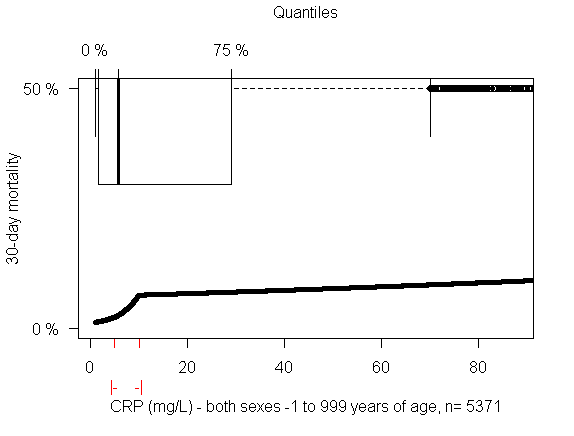
**

**Figure 7: The association between haemoglobin level and 30-day mortality among males presented as linear spline. Cuts are defined after recommended gender specific reference intervals (Male: 8.3 – 10.5 mmol/L. Female: 7.3 – 9.5 mmol/L). Top box-plot illustrates distribution of patients. Red lines mark the reference interval.**

**
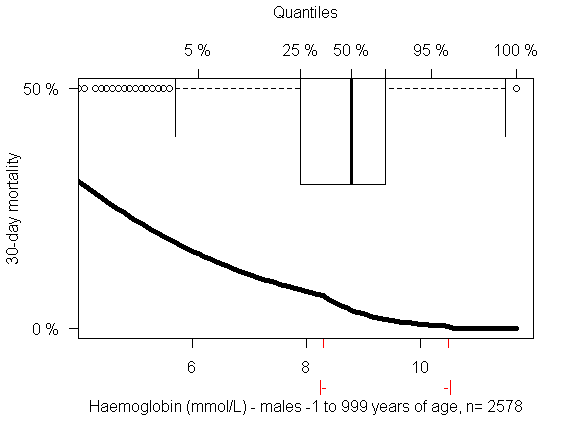
**

**Figure 8: The association between haemoglobin level and 30-day mortality among females presented as linear spline. Cuts are defined after recommended gender specific reference intervals (Male: 8.3 – 10.5 mmol/L. Female: 7.3 – 9.5 mmol/L). Top box-plot illustrates distribution of patients. Red lines mark the reference interval.**

**
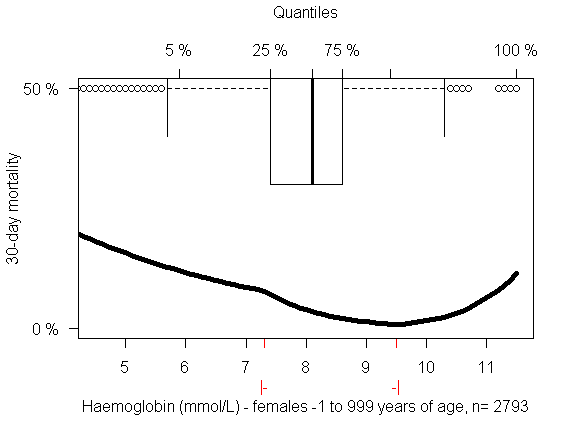
**

**Figure 9: The association between serum potassium concentration and 30-day mortality cumulated for both genders presented as linear spline. Cuts are defined after recommended reference interval (3.6-4.6 mmol/L). Top box-plot illustrates distribution of patients. Red lines mark the reference interval.**

**
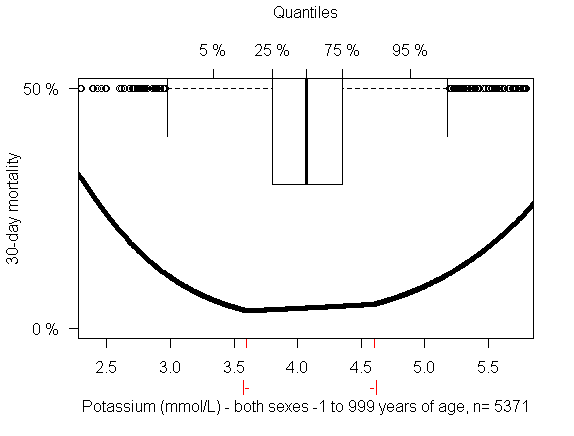
**

**Figure 10: The association between plasma creatinine concentration and 30-day mortality among females 18-70 years of age presented as linear spline. Cuts are defined after recommended age-gender specific reference intervals (Male: 60-105 µmol/L (60 - 125 µmol/L when >70 years). Female: 45-90 µmol/L (45 - 105 µmol/L when >70 years). Top box-plot illustrates distribution of patients. Red lines mark the reference interval.**

**
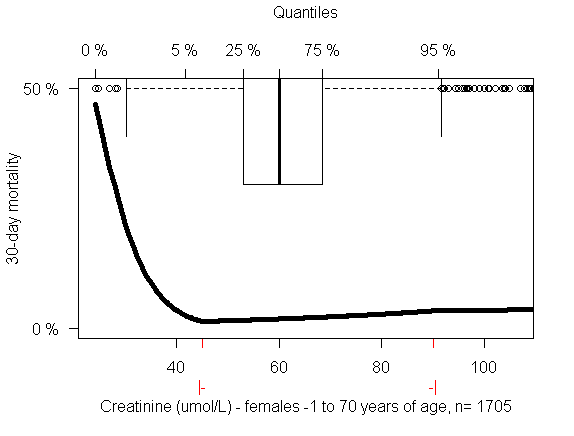
**

**Figure 11: The association between plasma creatinine concentration and 30-day mortality among females >70 years of age presented as linear spline. Cuts are defined after recommended age-gender specific reference intervals (Male: 60-105 µmol/L (60 - 125 µmol/L when >70 years). Female: 45-90 µmol/L (45 - 105 µmol/L when >70 years). Top box-plot illustrates distribution of patients. Red lines mark the reference interval.**

**
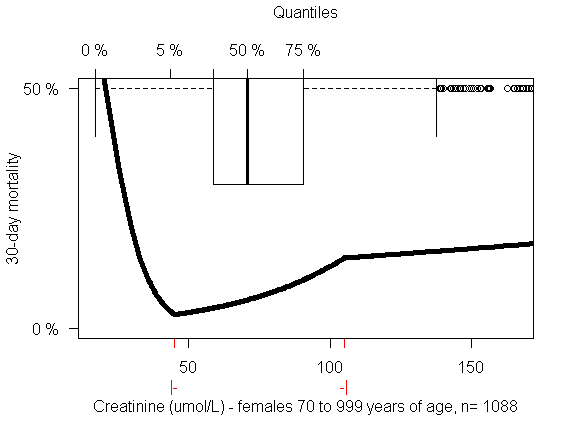
**

**Figure 12: The association between plasma creatinine concentration and 30-day mortality among males 18-70 years of age presented as linear spline. Cuts are defined after recommended age-gender specific reference intervals (Male: 60-105 µmol/L (60 - 125 µmol/L when >70 years). Female: 45-90 µmol/L (45 - 105 µmol/L when >70 years). Top box-plot illustrates distribution of patients. Red lines mark the reference interval.**

**
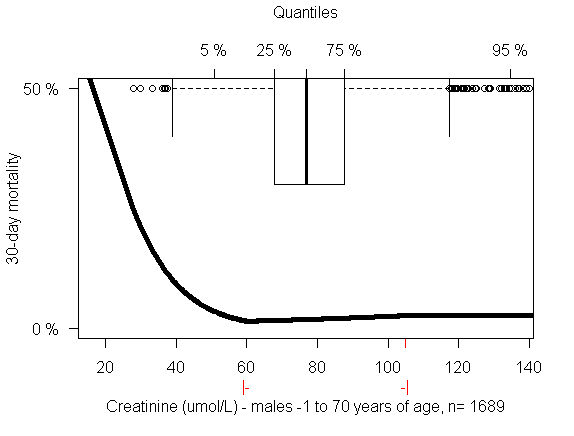
**

**Figure 13: The association between plasma creatinine concentratin and 30-day mortality among males >70 years of age presented as linear spline. Cuts are defined after recommended age-gender specific reference intervals (Male: 60-105 µmol/L (60 - 125 µmol/L when >70 years). Female: 45-90 µmol/L (45 - 105 µmol/L when >70 years). Top box-plot illustrates distribution of patients. Red lines mark the reference interval.**

**
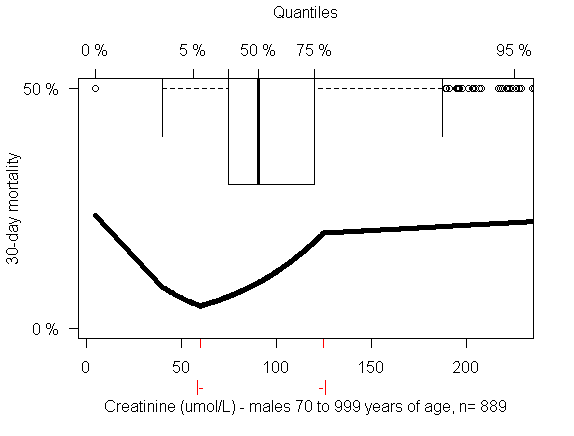
**

**Figure 14: The association between lactate dehydrogenase level and 30-day mortality among both genders 18-70 years of age presented as linear spline. Cuts are defined after recommended age specific reference intervals (< 70 years: 105-205 U/L . >70 years: 115-255 U/L). Top box-plot illustrates distribution of patients. Red lines mark the reference interval.**

**
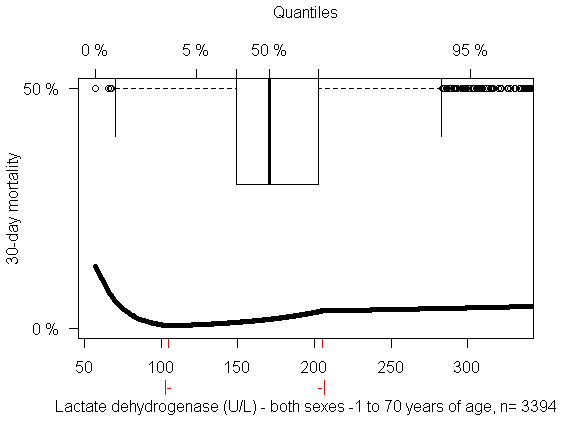
**

**Figure 15: The association between lactate dehydrogenase level and 30-day mortality among patients of both genders >70 years of age presented as linear spline. Cuts are defined after recommended age specific reference intervals (< 70 years: 105-205 U/L . >70 years: 115-255 U/L). Top box-plot illustrates distribution of patients. Red lines mark the reference interval.**

**
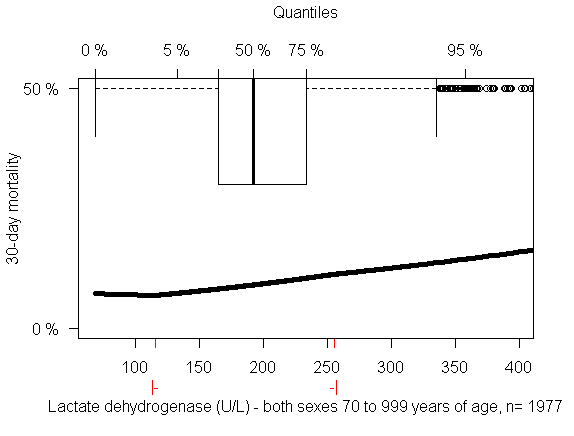
**
