# Supplementary material for: Routine blood tests are associated with short term mortality and can improve emergency department triage: a cohort study of >12,000 patients
Source: Scand J Trauma Resusc Emerg Med. 2017 Nov 28;25:115. doi: 10.1186/s13049-017-0458-x (PMC5704435; doi:10.1186/s13049-017-0458-x)
Supplement: Supplementary file 3 — Primary Cohort. Characteristics of included and excluded patients admitted to the emergency department in primary cohort (2010). (DOCX 16 kb) [file 13049_2017_458_MOESM3_ESM.docx]

# Primary Cohort

Characteristics of included and excluded patients admitted to the emergency department in primary cohort (2010).

|  | Included (n=5371) | Excluded (n=878) | p-value |
| --- | --- | --- | --- |
| Age (median, IQR) | 63.8 [46.9 - 76.5] | 57.0 [38.1 - 71.7] | <0.001 |
| Male gender (n, %) | 2578 (48.0) | 435 (49.5) | 0.42 |
| Survival > 30 days (n, %) | 284 (5.3) | 44 (5.0) | 0.80 |
| Intensive care unit admission (n, %) | 85 (1.6) | 26 (3.0) | <0.01 |
| Length of stay, days (median, IQR) | 2 [1 - 4] | 1 [1 - 2] | <0.001 |
| Readmission within 30 days (n, %) | 760 (14.2) | 121 (13.8) | 0.81 |
| Admission during weekend (n, %) | 1235 (23.8) | 211 (27.1) | 0.05 |
| Arrival between 8 pm and 6 am (n, %) | 1393 (26.9) | 261 (33.5) | <0.001 |
| Arterial oxygen saturation, % (median, IQR) | 98 [96 - 99] | 98 [97 - 99] | 0.03 |
| Respiratory rate, min^-1^ (median, IQR) | 16 [16 - 20] | 16 [14 - 20] | <0.001 |
| Heart rate, min^-1^ (median, IQR) | 82 [71 - 95] | 81 [71 - 92] | 0.08 |
| Systolic blood pressure, mmHg (median, IQR) | 140 [125 - 157] | 139 [124 - 157] | 0.64 |
| Glasgow coma scale (median, IQR ) | 15 [15 - 15] | 15 [15 - 15] | 0.76 |
| C-reactive protein, nmol/L (median, IQR) | 5.8 [1.7 - 29.1] | 3.5 [1.4 - 17.2] | 0.59 |
| Potassium, mmol/L (median, IQR) | 4.1 [3.8 - 4.4] | 4.1 [3.8 - 4.4] | 0.49 |
| Sodium, mmol/L (median, IQR) | 137.5 [135 - 139.4] | 137.2 [134 - 139.6] | 0.41 |
| Haemoglobin, mmol/L (median, IQR) | 8.4 [7.6 - 9.1] | 8.4 [7.6 - 9.2] | 0.50 |
| Creatinine, μmol/L (median, IQR) | 71.2 [59.0 - 87.0] | 73.0 [59.5 - 88.6] | 0.02 |
| Leukocyte count, 109/L (median, IQR) | 8.69 [6.8 - 11.5] | 9.6 [7.2 - 12.4] | <0.01 |
| Albumin, g/L (median, IQR) | 42.0 [38.6 - 44.7] | 41.4 [36.3 - 44.2] | <0.01 |
| Lactate dehydrogenase, U/L (median, IQR) | 178.1 [153.3 - 213.6] | 203.8 [166.4 - 233.5] | 0.29 |
