# Supplementary material for: Routine blood tests are associated with short term mortality and can improve emergency department triage: a cohort study of >12,000 patients
Source: Scand J Trauma Resusc Emerg Med. 2017 Nov 28;25:115. doi: 10.1186/s13049-017-0458-x (PMC5704435; doi:10.1186/s13049-017-0458-x)
Supplement: Supplementary file 4 — Cohort Characteristics. Characteristics of the primary cohort (2010) and the validation cohort (2013). (DOCX 17 kb) [file 13049_2017_458_MOESM4_ESM.docx]

# Cohort characteristics

**Characteristics of the primary cohort (2010) and the validation cohort (2013).**

| Primary cohort (2010) | Validation cohort (2013) |
| --- | --- |
| **Time:**  2010 | **Time:**  2013  **Duration:**  3.5 months  **Location:**  Nordsjælland University Hospital  Uptake area size: 310.000 citizens  Yearly emergency department contacts: 68.000  **Included patients:**  5738 of 6383 (89.9%)  **30 day mortality:**  4.1% |
| **Duration:**  5 months |  |
| **Location:**  Nordsjælland University Hospital  Uptake area size: 310.000 citizens  Yearly emergency department contacts: 50.000 |  |
| **Included patients:**  5371 of 6279 (85.5%) |  |
| **30 day mortality:**  5.3% |  |
|  |  |
