# Supplementary material for: Routine blood tests are associated with short term mortality and can improve emergency department triage: a cohort study of >12,000 patients
Source: Scand J Trauma Resusc Emerg Med. 2017 Nov 28;25:115. doi: 10.1186/s13049-017-0458-x (PMC5704435; doi:10.1186/s13049-017-0458-x)
Supplement: Supplementary file 5 — Recalibration. Notes on the recalibration process. Including table depicturing reclassification of patients by the blood test prediction model and Kaplan Meier survival probabilities. (DOCX 38 kb) [file 13049_2017_458_MOESM5_ESM.docx]

# Recalibration

Calibration of the model when initially applied on the validation cohort was poor. Recalibration was therefore performed using logistic calibration as described by Janssen et al. 2008 to obtain accurate prediction.^27^

Table 1 shows distribution of patients in the four risk strata according to Formalized triage (HAPT or DEPT) and prediction model (30-day mortality in parenthesis). Table 1A shows risk stratification on the primary cohort. Note the large number of low risk patients (“green” patients) with a significantly lower mortality (0.1% [95%CI 0.0;0.3%]) compared to formalized HAPT triage (2.8% [95%CI 2.0;3.6%]).

Table 1B shows risk stratification on validation cohort. Directly applied on the validation cohort, the prediction model seemed to overestimate the risk of 30-day mortality illustrated by a larger number of patients in the high risk groups and fewer in the low risk groups. Recalibration of the model yielded a predictive ability equivalent to in the primary cohort. Figure 1 illustrates this reclassification in Kaplan-Meier plots with 95% confidence intervals.

**Table 1: Distribution of patients by formalized triage and blood test prediction model.**

| A | Primary cohort (n=5,371) | |
| --- | --- | --- |
|  | HAPT | Blood test prediction^a^ |
| Green | 1591 (2.8% [2.0;3.6]) | 2100 (0.1% [0.0;0.3]) |
| Yellow | 2174 (4.6% [3.7;5.4]) | 2533 (3.8% [3.1;4.6]) |
| Orange | 1447 (7.2% [5.9;8.5]) | 501 (16.0% [12.8;19.2]) |
| Red | 159 (22.6% [16.1;29.1]) | 237 (43.9% [37.6;50.2]) |

| B | Validation cohort (n=5,738) | | |
| --- | --- | --- | --- |
|  | DEPT | Blood test prediction^a^  No calibration | Blood test prediction^a^  Recalibrated |
| Green | 1876 (2.1% [1.4;2.7]) | 235 (0% [0;0]) | 2030 (0.4% [0.1;0.7]) |
| Yellow | 2272 (3.9% [3.1;4.7]) | 4205 (1.3% [0.9;1.6]) | 3174 (3.3% [2.7;3.9]) |
| Orange | 1557 (6.0% [4.9;7.2]) | 821 (8.0% [6.2;9.9]) | 365 (15.1% [11.4;18.7%]) |
| Red | 33 (36.4%[20.0;52.8%]) | 477 (23.9% [20.1;27.7]) | 169 (39.6% [32.3;47.0]) |

Distribution of patients, risk stratified by formalized triage algorithm (HAPT or DEPT) or the blood test prediction model in primary (A) and validation cohort (B). 30-day mortality with 95% confidence intervals for individual strata in parenthesis. For the validation cohort prediction model reclassification is shown before and after recalibration.

a: Predicted risk of 30-day mortality: green <1%, yellow 1-10%, orange 10-25%, red >25%

**Figure 1. Kaplan-Meier survival probabilities stratified on triage and the blood test prediction model.**

**
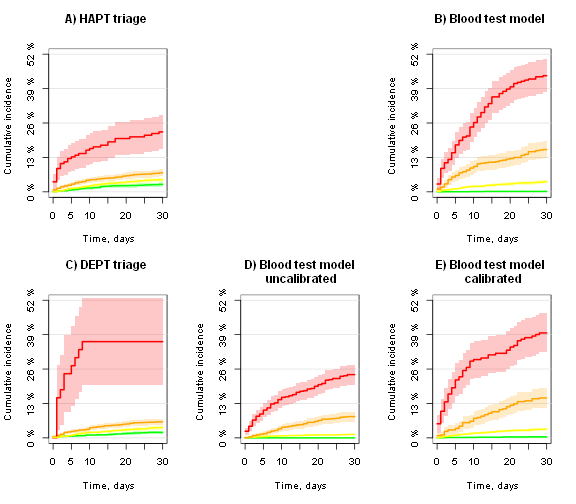
**

Cumulative percentage of mortality within 30 days for triage and blood test prediction model in patients presenting in the emergency department. “Green”, “yellow”, “orange” and “red” patients are plotted with 95% confidence intervals. A + B: HAPT triage and blood test prediction model on primary cohort. C + D + E: DEPT triage and blood test prediction model before and after recalibration on validation cohort
